# IFN-γ regulates human dental pulp stem cells behavior via NF-κB and MAPK signaling

**DOI:** 10.1038/srep40681

**Published:** 2017-01-18

**Authors:** Xinyao He, Wenkai Jiang, Zhirong Luo, Tiejun Qu, Zhihua Wang, Ningning Liu, Yaqing Zhang, Paul R. Cooper, Wenxi He

**Affiliations:** 1 State Key Laboratory of Military Stomatology, Department of Operative Dentistry & Endodontics, School of Stomatology, the Fourth Military Medical University, Xi’an Shaanxi, China; 2 Shaanxi Key Laboratory of Stomatology, School of Stomatology, the Fourth Military Medical University, Xi’an Shaanxi, China; 3 Department of Stomatology, the Affiliated Hospital of Guizhou Medical University, Guiyang Guizhou, China; 4 Oral Biology, School of Dentistry, University of Birmingham, B4 6NN, UK

## Abstract

During caries, dental pulp expresses a range of pro-inflammatory cytokines in response to the infectious challenge. Interferon gamma (IFN-γ) is a dimerized soluble cytokine, which is critical for immune responses. Previous study has demonstrated that IFN-γ at relative high concentration (100 ng/mL) treatment improved the impaired dentinogenic and immunosuppressive regulatory functions of disease-derived dental pulp stem cells (DPSCs). However, little is known about the regulatory effects of IFN-γ at relative low concentration on healthy DPSC behavior (including proliferation, migration, and multiple-potential differentiation). Here we demonstrate that IFN-γ at relatively low concentrations (0.5 ng/mL) promoted the proliferation and migration of DPSCs, but abrogated odonto/osteogenic differentiation. Additionally, we identified that NF-κB and MAPK signaling pathways are both involved in the process of IFN-γ-regulated odonto/osteogenic differentiation of DPSCs. DPSCs treated with IFN-γ and supplemented with pyrrolidine dithiocarbamate (PDTC, an NF-κB inhibitor) or SB203580 (a MAPK inhibitor) showed significantly improved potential for odonto/osteogenic differentiation of DPSCs both *in vivo* and *in vitro*. These data provide important insight into the regulatory effects of IFN-γ on the biological behavior of DPSCs and indicate a promising therapeutic strategy for dentin/pulp tissue engineering in future endodontic treatment.

Dental caries is a chronic infectious disease, which can lead to the demineralization of enamel and dentin and subsequent pulp tissue injury^1^. Pulpitis is an inflammatory disease which occurs when the infection of caries-related microorganisms penetrates the dentinal tubules and into the pulpal tissue^2^. During the inflammatory phase, the pulp tissue reacts to bacterial irritants via innate and/or adaptive immune responses, thus releasing a range of chemokines and pro-inflammatory cytokines^3^. Inflammation and regeneration in dental pulp tissue can appear to be contradictory processes, however, they are clearly inter-related^4^. The pro-inflammatory cytokines released from the inflammatory milieu play a crucial role in the process of pulp tissue healing and dentin regeneration through synchronized regulation of the behavior of inflammatory and stem/progenitor cells^5^,^6^.

Dental pulp stem cells (DPSCs) are mesenchymal-derived cells characterised by self-renewal and multi-lineage differentiation (including chondrocyte, adipocyte, neural and osteoblast lineage differentiation), establishing them as an attractive choice for tissue engineering and regenerative medicine purposes^7^. Numerous studies have demonstrated that DPSCs have the potential to generate dentin and pulp tissues under appropriate environmental conditions^8^,^9^. In pulpitis, DPSCs can form tertiary dentin adjacent to the injury site by proliferation, migration and differentiation into odontoblast-like cells^10^. A variety of pro-inflammatory cytokines which alter the biological behavior (including proliferation, migration, differentiation) of DPSCs, reportedly play vital physiological roles in regeneration of pulp and dentin. For instance, short-term treatment with TNF-α promotes the proliferation, migration, and multipotent differentiation of DPSCs^11^. Additionally, orthodontic treatment enhanced the differentiation and self-renewal of DPSCs in a rat model, mediated through orthodontic-induced inflammation and subsequent elevation of IL-17A levels with the dental pulp microenvironment^12^.

Interferon gamma (IFN-γ) is a dimerized soluble cytokine, which is critical for innate and adaptive immune responses targeted against viral, bacterial and protozoal infections^13^. In contrast with several studies which describe the role of IFN-γ in the immune responses, recent work has now shown that IFN-γ is required for the osteogenic differentiation of mesenchymal stem cells (MSCs)^14^, as well as neuronal differentiation of neural stem cells^15^. Recently, studies have focused increasingly on the roles of IFN-γ in DPSCs. It has been reported that IFN-γ at relative high concentration (100 ng/mL) treatment improved the impaired dentinogenic and immunosuppressive regulatory functions of disease-derived DPSCs following pulpitis^16^. While DPSCs provide a potential source of progenitor cells for dentin regeneration, the mechanistic basis for the action of IFN-γ at relative low concentration in the regulation of healthy DPSCs behavior remains unclear. Therefore, the aim of this study was to investigate the effect of IFN-γ on the proliferation, migration and odonto/osteogenic differentiation of healthy DPSCs, as well as determining the underlying signaling pathway(s) associated with IFN-γ-regulated DPSC behavior *in vitro*. Furthermore, we explored the co-application of DPSCs and IFN-γ onto nanofibrous gelatin (NF-gelatin) scaffolds to investigate dentin regeneration *in vivo* and the associated signaling pathways.

## Results

### Isolation and characterization of DPSCs

**Figure 1:**
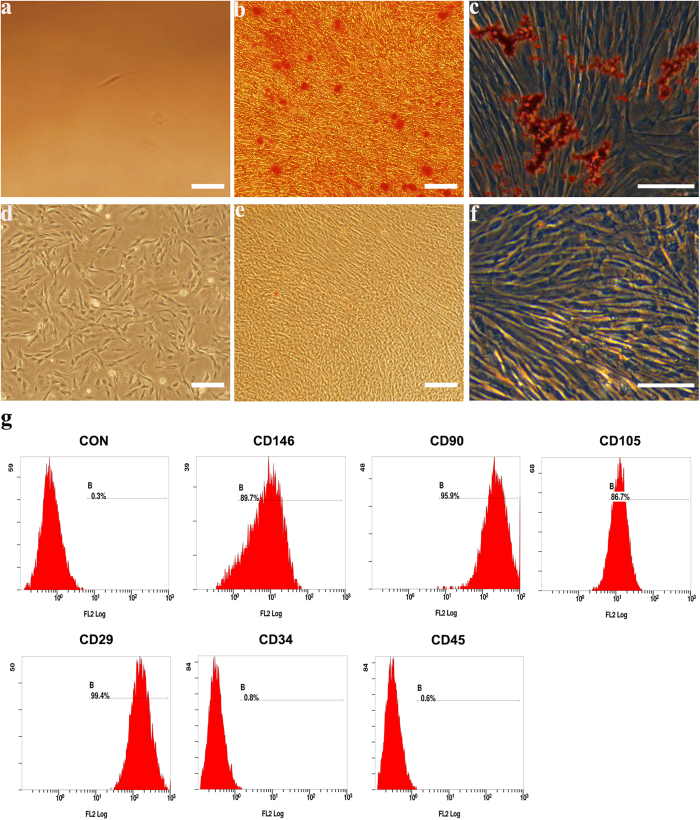
Characterization of human dental pulp stem cells (DPSCs). The fibroblast-like monoclonal cell isolated from primary cells (**a**) monoclonal cells at day 14 (**d**). This clone was able to differentiate into the mesenchymal lineages: osteogenic (alizarin red staining) (**b**) adipogenic (oil-red o staining) (**c**) when cultured in appropriate differentiation conditions *in vitro* compared to control groups (**e**,**f**), respectively. Analysis of molecular surface antigen markers in DPSCs by flow cytometry indicated that cells were negative for CD34 and CD45, whereas they were positive for CD146, CD90, CD105, CD29; PE-conjugated non-specific mouse IgG1 served as negative controls (**g**). Bar 100 μm.

Dental pulp cells were successfully isolated from pulp tissue of extracted third molars. The primary cells presented clone-like growth after they were incubated for 72 h. The fibroblast-like monoclonal cell was isolated by limiting dilution (Fig. 1a). Putative stem cells obtained from the cell clones that were grown for 14 days (Fig. 1d) were characterized by multiple lineage differentiation tests and flow cytometric. The multiple lineage differentiation tests revealed that the cells stained positive for mineral nodules with alizarin red staining after 3 weeks of osteogenic induction (Fig. 1b). After 5 weeks of adipogenic induction, the cells stained positive for lipid droplets with oil-red o staining (Fig. 1c). Flow cytometric analysis revealed that these cells were negative for CD34 and CD45. The cultures population contained 89.7% CD146-positive cells, 95.9% CD90-positive cells, 86.7% CD105-positive cells, 99.4% CD29- positive cells (Fig. 1g). In addition to their morphology, colony-forming ability, as well as the expression of specific antigens on the cell surface and the capacity for differentiation into different mesenchymal tissues suggests that the cells that we obtained have the key properties of DPSCs.

### IFN-γ stimulates the proliferation of DPSCs

**Figure 2:**
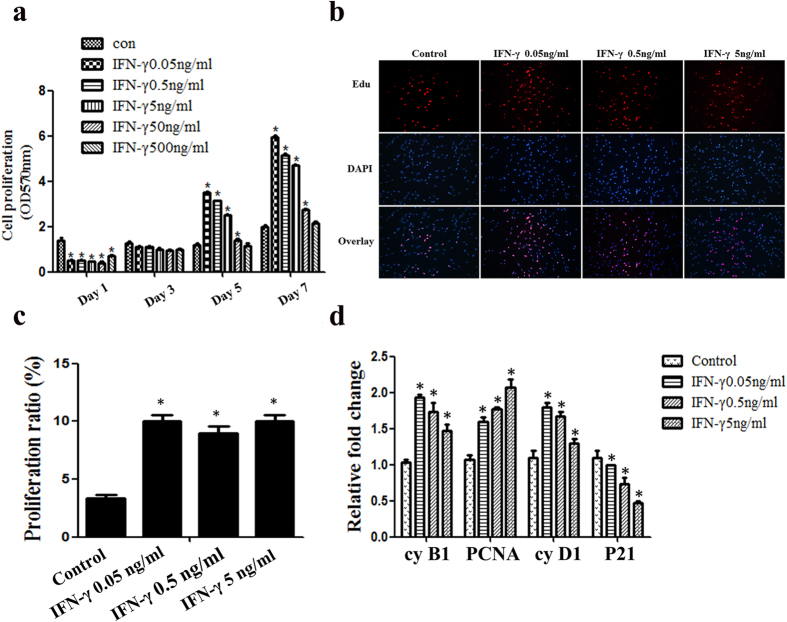
The effects of IFN-γ on the proliferative capacity of DPSCs. DPSCs were treated with IFN-γ at different concentrations for the indicated time. Cell viability was evaluated using the MTT (**a**) and EdU incorporation assays (**b**,**c**). Scale bars = 50 μm. The mRNA expression of cyclin B1, PCNA, cyclin D1 and P21 was analysed by Q-PCR (**d**). Statistical analysis was performed using one-way ANOVA. Data are shown as the mean ± SD. *P < 0.05 when compared with the control group.

The proliferation rates of DPSCs were analysed in response to increasing doses of IFN-γ using the MTT and EdU-incorporation assays. IFN-γ significantly suppressed the growth of DPSCs at the early stages of cell culture. However, IFN-γ at lower concentrations (including 0.05 ng/mL, 0.5 ng/mL and 5 ng/mL) significantly promoted the proliferation of DPSCs after day 5 and this effect persisted to day 7 (Fig. 2a). Consistently, the EdU-incorporation assay showed that IFN-γ at concentrations of 0.05 ng/mL, 0.5 ng/mL and 5 ng/mL also significantly promoted the proliferation rate of DPSCs and no significant differences were observed between the experimental groups (Fig. 2b,c).

To further corroborate our observation, Q-PCR was performed to analyse the mRNA expression of cell cycle related proteins. The cell cycle promoting molecules (cyclin B1, cyclin D1, PCNA) were up-regulated, whilst those negatively regulating cell cycle progression (P21) were down-regulated in response to the lower concentrations of IFN-γ (Fig. 2d). Based on the above data, we therefore applied IFN-γ at concentrations of 0.05 ng/mL, 0.5 ng/mL and 5 ng/mL in subsequent assays.

### IFN-γ enhances the migration of DPSCs

**Figure 3:**
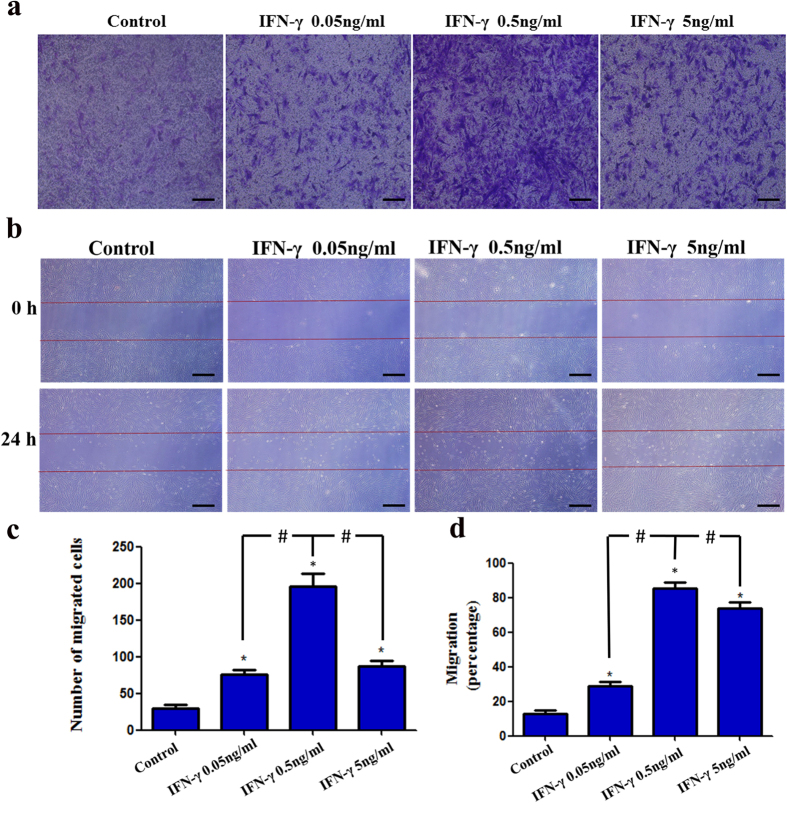
The effects of IFN-γ on the migration of DPSCs. Cell migration was evaluated with a two-chamber Transwell system. The migratory cells were stained with crystal violet (**a**). For the wound healing assay, cells were incubated with IFN-γ at different concentrations for 24 h. Photomicrographs of the scratch were taken at 0 and 24 h post wounding (**b**). Statistical analysis was performed using one-way ANOVA. Data are shown as the mean ± SD. *P < 0.05 when compared with the control group. ^#^P < 0.05 when compared with the IFN-γ 0.5 ng/mL group. Scale bars = 100 μm.

To determine whether IFN-γ could regulate the migration of DPSCs, transwell migration and wound healing assays were performed. Results indicated that IFN-γ at concentrations of 0.05 ng/mL, 0.5 ng/mL and 5 ng/mL increased the motility of DPSCs. In particular, 0.5 ng/mL IFN-γ significantly increased the number of migratory cells compared with the 0.05 ng/mL and 5 ng/mL groups (Fig. 3a,c). In parallel, the wound healing assay also showed that 0.5 ng/mL IFN-γ significantly decreased the wound gap compared with either the control group or other experimental groups (Fig. 3b,d). These findings suggest that IFN-γ (especially at a concentration of 0.5 ng/mL) promotes the migration of DPSCs.

### IFN-γ inhibits the odonto/osteogenic differentiation of DPSCs *in vitro*

**Figure 4:**
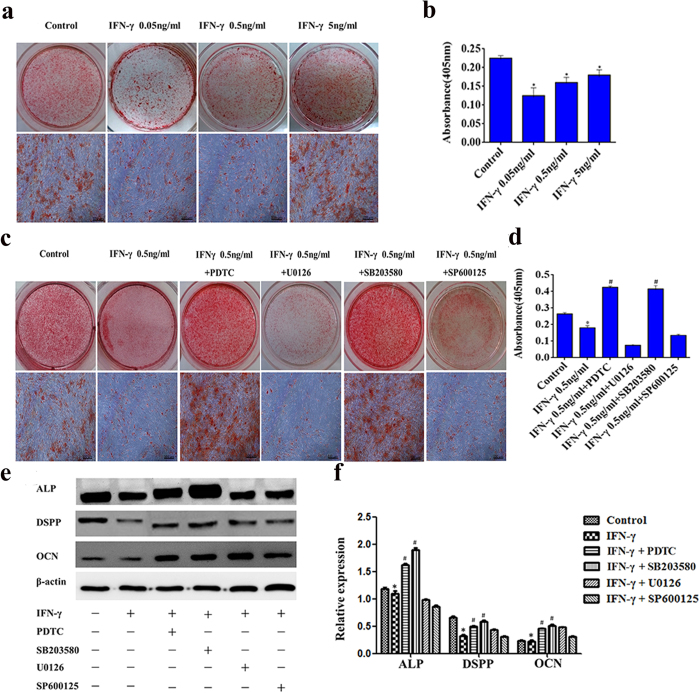
The effects of IFN-γ on the odonto/osteogenic differentiation of DPSCs. DPSCs were cultured in odonto/osteogenic medium containing IFN-γ at different concentrations for 2 weeks with or without the MAPK inhibitor (SB203580: 20 μΜ, U0126: 25 μΜ, SP600125: 25 μΜ), or NF-κB inhibitor (PDTC: 20 μΜ). Alizarin red staining and alizarin red quantification were used to evaluate the formation of calcium nodules (**a**–**d**). The protein expression of ALP, DSPP, OCN and β-actin was analysed by Western blotting (**e**, Supplementary Fig. 1a). The relative band intensities were determined by densitometry (**f**). Statistical analysis was performed using one-way ANOVA. Data are shown as the mean ± SD. *P < 0.05 when compared with the control. ^#^P < 0.05 when compared with the IFN-γ group. Scale bars = 100 μm.

To evaluate the effects of IFN-γ on the odonto/osteogenic differentiation of DPSCs, DPSCs were cultured in mineralization medium and challenged with IFN-γ, prior to alizarin red staining. After 2 weeks incubation, the number of mineralized nodules was significantly increased in mineralization medium; however, when the cells were treated with IFN-γ at concentrations of 0.05 ng/mL, 0.5 ng/mL or 5 ng/mL, the number of nodules significantly diminished (Fig. 4a,b). Additionally, the protein expression for alkaline phosphatase (ALP), dentin sialophosphoprotein (DSPP) and osteocalcin (OCN) was analysed by western blotting. Compared with the control group, 0.5 ng/mL IFN-γ significantly down-regulated the protein expression of ALP, DSPP and OCN indicating that exposure to IFN-γ inhibits the odonto/osteogenic differentiation of DPSCs (Fig. 4e,f).

To further investigate signaling pathways, DPSCs were treated with mineralization medium containing IFN-γ 0.5 ng/mL, in the presence or absence of MAPK inhibitors (SB203580, U0126, and SP600125) or the NF-κB inhibitor (pyrrolidine dithiocarbamate, PDTC) for 2 weeks. The results of alizarin red staining showed that the presence of SB203580 or PDTC markedly increased the formation of mineralization nodules compared with the IFN-γ alone group. However, the co-stimulation with U0126 or SP600125 had minimal effect on the formation of mineralization nodules compared with the IFN-γ alone group. Quantitative measurement of alizarin red S staining confirmed the above results (Fig. 4c,d). Consistently, the inhibition with PDTC or SB203580 triggered an increase in the protein expression of ALP, DSPP and OCN, compared with the IFN-γ alone group. (Fig. 4e,f).

### Involvement of the NF-κB and MAPK signaling pathways in the IFN-γ-induced inhibition of DPSCs odonto/osteogenic differentiation

**Figure 5:**
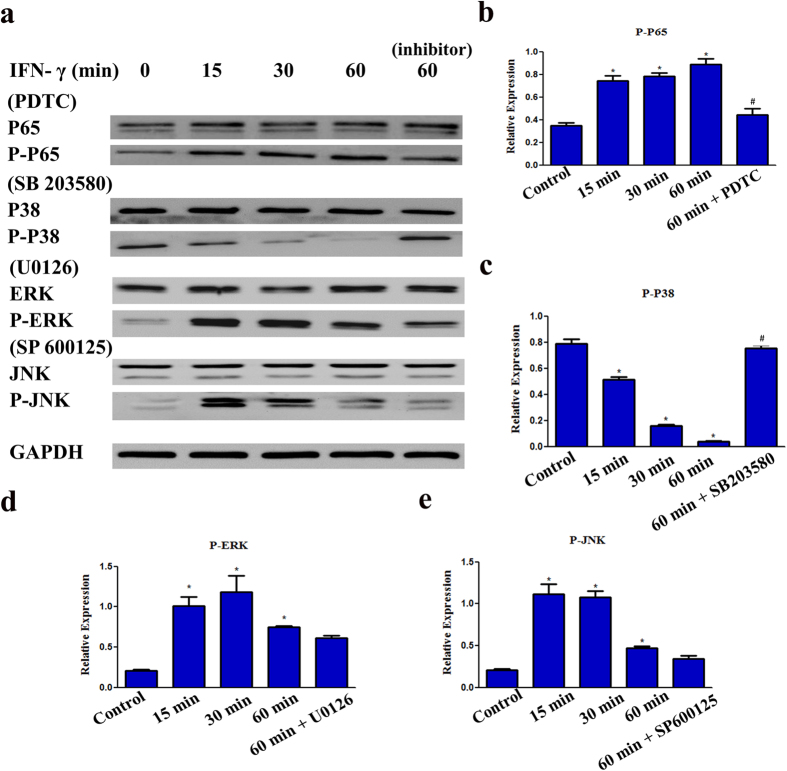
Involvement of NF-κB and MAPK signaling pathways in the IFN-γ-induced inhibition odonto/osteogenic differentiation of DPSCs. DPSCs were cultured in odonto/osteogenic medium containing 0.5 ng/ml IFN-γ with or without MAPK inhibitor (SB203580: 20 μΜ, U0126: 25 μΜ, SP600125: 25 μΜ), or NF-κB inhibitor (PDTC: 20 μΜ). The protein expression of P65, p-P65, P38, p-P38, ERK, p-ERK, JNK, p-JNK was analysed by Western blotting (**a**, Supplementary Fig. 1b). The relative band intensities were determined by densitometry (**b**–**e**). Statistical analysis was performed by using one-way ANOVA. Data are shown as means ± SD. *P < 0.05 when compared with the control group. ^#^P < 0.05 when compared with the IFN-γ 60 min group.

To further identify whether the NF-κB signaling pathway is involved in the IFN-γ mediated inhibition odonto/osteogenic differentiation of DPSCs, we evaluated the NF-κB signaling protein P65 by western blotting. The phosphorylation of P65 (p-P65) was up-regulated by IFN-γ in a time-dependent manner. However, pharmacological inhibition of NF-κB signaling by PDTC partially down-regulated p-P65 compared with the 60 min group, which indicates that the P65 NF-κB signaling pathway is involved in the IFN-γ mediated inhibition odonto/osteogenic differentiation of DPSCs (Fig. 5a,b).

Protein levels of phosphorylated P38 (p-P38) were down-regulated by IFN-γ in a time-dependent manner, and reached a minimum after 60 min, whist pharmacological inhibition of MAPK signaling by SB203580 significantly up-regulated p-P38 compared with the 60 min group (Fig. 5a,c). Additionally, with IFN-γ treatment, the protein levels of p-ERK and p-JNK appeared to rise after 15 min and reached a peak at 30 min, but subsequently decreased after 60 min treatment (Fig. 5a,d,e). U0126 and SP600125 inhibition exerted a minimal effect on the protein expression of p-ERK and p-JNK compared with the 60 min group (Fig. 5a,d,e).

### IFN-γ impairs the odonto/osteogenic differentiation of hDPSCs *in vivo*

**Figure 6:**
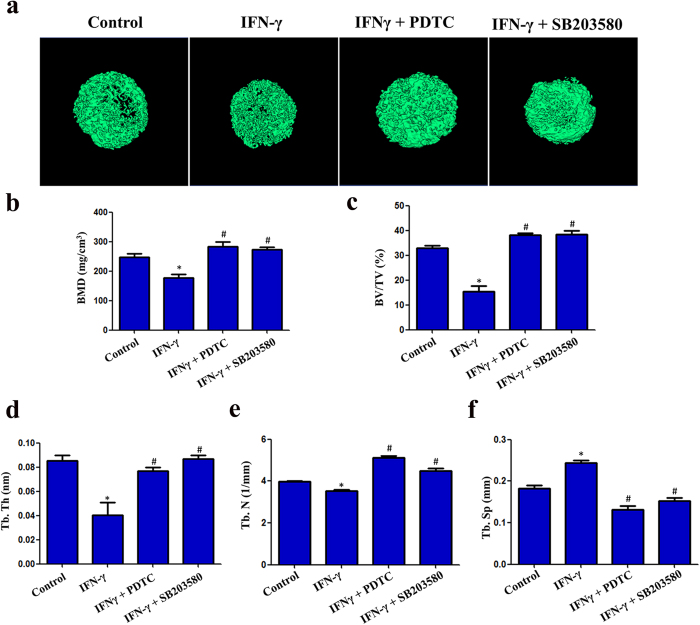
Micro-computed tomographic (CT) analyses of bone structural and mineral density changes. Three dimensional reconstruction images from micro-CT analysis (**a**). IFN-γ decreased the BMD, BV/TV, Tb. Th and the Tb. N parameters, but increased the Tb. Sp parameter (**b**–**f**). TNF-κB inhibitor (PDTC) and MAPK inhibitor (SB203580) reversed the effects induced by IFN-γ in DPSCs *in vivo*. (BMD: bone mineral density; BV/TV: bone volume/total volume; Tb. Th: trabecular thickness; Tb. N: trabecular number; Tb. Sp: trabecular separation). *P < 0.05 when compared with the control group. ^#^P < 0.05 when compared with the IFN-γ group. (n = 4 for each group).

Micro-CT reconstructed images for the different groups are shown in Fig. 6a. In the IFN-γ group, the trabecular separation (Tb. Sp) was increased (Fig. 6f) while the bone mineral density (BMD) (Fig. 6b), bone volume/total volume (BV/TV) (Fig. 6c), trabecular thickness (Tb. Th) (Fig. 6d), and trabecular number (Tb. N) (Fig. 6e) were decreased compared with the control group. IFN-γ co-treatment with inhibitors (PDTC or SB203580) significantly restored the reductions in BMD, BV/TV, Tb. Th and Tb. N (Fig. 6b–e), but decreased the Tb. Sp (Fig. 6f), reflecting the improved odonto/osteogenic potential of the DPSCs under these conditions.

**Figure 7:**
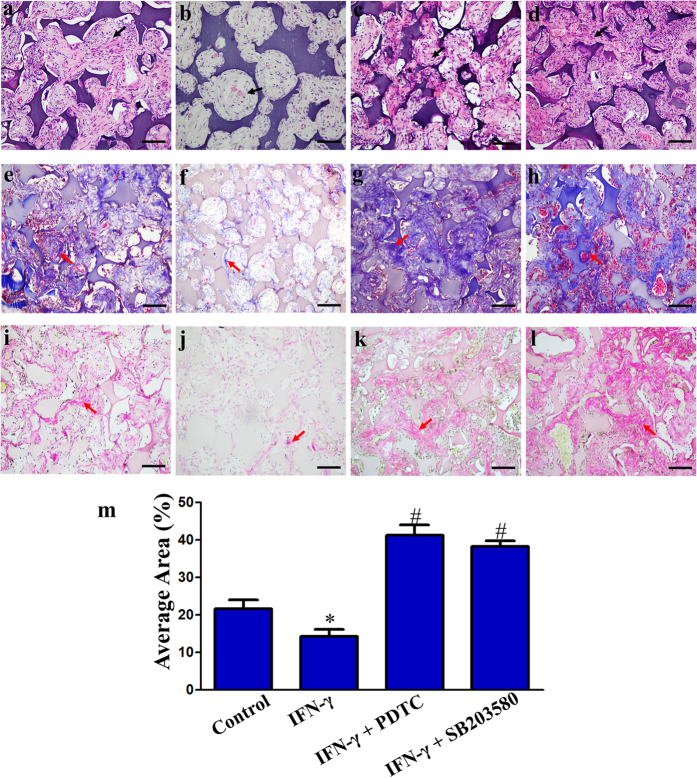
Histological analyses of dental pulp stem cells (DPSCs) seeded on NF-gelatin scaffolds after 4 weeks of ectopic *in vivo* implantation in immunodeficient mice. Histology of control cell-scaffold constructs (**a**,**e**,**i**), IFN-γ treated cell-scaffold constructs (**b**,**f**,**j**), cell-scaffold constructs co-stimulated with IFN-γ and PDTC (**c**,**g**,**k**) and cell-scaffold constructs co-stimulated with IFN-γ and SB203580 (**d**,**h**,**l**). H&E staining (**a**–**d**), Masson’s trichrome staining (**e**–**h**), Van Gieson staining (**i**–**l**). Scale bars = 100 μm. Black arrows: the newly formed ECM-like architecture. Red arrows: mineralized tissue consisting of newly formed dentin-like matrix. Quantitative analyses of the newly formed dentin-like matrix areas quantified using Image-Pro Plus 6.0 software (**m**). *P < 0.05 when compared with the control group. ^#^P < 0.05 when compared with the IFN-γ group. (n = 4 for each group).

DPSCs penetrated into each pore of the NF-gelatin scaffold. Newly formed extracellular matrix (ECM)-like architecture (Fig. 7 black arrow) and dentin-like matrix (Fig. 7 red arrow) were in direct contact with the scaffold. Masson’s trichrome and Van Gieson staining revealed a significantly higher amount of dentin-like matrix in the NF-gelatin groups compared to the IFN-γ induced group (Fig. 7e,f and i,j). Quantitative image analysis revealed that the total area of newly formed dentin-like matrix was significantly diminished in the IFN-γ induced group compared with the control group (P < 0.05) (Fig. 7m). While treatment with the NF-κB inhibitor (PDTC) or MAPK inhibitor (SB203580) markedly increased the total area of newly formed dentin-like matrix compared with the IFN-γ induced group, indicating an improved dentinogenic potential of the DPSCs (Fig. 7e–m).

## Discussion

Dental caries or trauma can give rise to an inflammatory response in the dental pulp. Gram-negative bacteria (e.g. *Fusobacterium, Prevotella*, and *Porphyromonas*) has been reported to be closely associated with symptomatic teeth, infected pulps and periapical abscesses^17^,^18^. Lipopolysaccharides (LPS), a major component of the membrane of Gram-negative bacteria, has been shown to be responsible for pulp infection and can trigger a protective inflammatory response which is characterised by release of a range of inflammatory cytokines, including interleukins (IL-1α, IL-1β, and IL-6), TNF-α, and IFN-γ^11^. In our previous study, we have demonstrated that LPS treatment significantly promoted the odontoblastic differentiation of DPSCs in a dose-dependent manner^19^. However, the effects of inflammatory cytokines induced by LPS on the regulation of biological behaviors of DPSCs remains unclear. Numerous studies have reported that these cytokines can also have significant impact on the behavior of progenitor/stem cells in the pulp, leading to deposition of reparative tertiary dentin in response to the injury. Among these cytokines, IFN-γ represents a higher prevalence in shallow caries and correlated with the presence of *Streptococcus mutans*, which suggests that it has a major impact on the initial lesion and regeneration of reparative tertiary dentin. Therefore, we herein analysed the effect of IFN-γ on regulation of DPSCs biological behaviors, including proliferation, migration and odonto/osteogenic differentiation.

Previous studies have demonstrated that TNF-α alone stimulates proliferation of human DPSCs via Akt/Glycogen Synthase Kinase-3β/Cyclin D1 signaling pathways^20^. Additionally, TNF-α together with IFN-γ increased the proliferation of murine adipose tissue-derived MSCs^21^. In this study, we found that high concentrations of IFN-γ exerted little effect on the proliferation of DPSCs, which is consistent with data previously reported^22^. In the present study, we have found that IFN-γ at relatively low concentrations promoted the proliferation of DPSCs in a time-dependent manner. To further investigate the IFN-γ-induced proliferation of DPSCs, we evaluated the gene expression of cell proliferation-associated markers, including proliferating cell nuclear antigen (PCNA), Ki-67 antigen, Cyclin B1, Cyclin D1 and P21^23^. PCNA and Ki-67 are nuclear proteins preferentially expressed in cycling but not in resting cells^24^,^25^. The observed up-regulation of PCNA and Ki-67 indicates the high percentage of cells in a cycling state. Cyclins are a family of proteins that regulate the progression of cells through the cell cycle^26^. P21, a potent cyclin-dependent kinase inhibitor, functions as a negative regulator of cell cycle progression^27^. The expression of proteins promoting the cell cycle (Cyclin B1, Cyclin D1, PCNA) was up-regulated, whist a protein negatively regulating cell cycle progression (P21) was down-regulated. These data confirm that low concentration of IFN-γ promoted proliferation of DPSCs.

The healing process of an injured tissue can be considered to comprise of four phases, including hemostasis, inflammation, proliferation, and remodeling, which are not independent, but synergistic^28^. During the inflammatory period, the pro-inflammatory cytokines released by activated immune cells play an important role not only in recruiting neutrophils to inflammatory sites, but also in activation of surrounding connective tissue cells, including stem/progenitor cells^29^,^30^. These stem cells proliferate and migrate to the injury sites, thus contributing to tissue healing through direct differentiation or indirect paracrine stimulation of endogenous reparative responses. Therefore, pro-inflammatory cytokine-induced stem cell migration is a key step in the healing process. The data above demonstrated that low concentrations of IFN-γ promoted the proliferation of DPSCs. We then further demonstrated that the same concentrations of IFN-γ (especially at a concentration of 0.5 ng/mL) also promoted the migration of DPSCs. Previous studies have reported that IFN-γ effectively promotes the migration of MSCs derived from human umbilical cord and bone marrow during tissue regeneration^31^. We herein have provided evidence of IFN-γ-induced DPSC migration directly associated with dentin/pulp regeneration.

Pro-inflammatory cytokine-induced odonto/osteogenic differentiation of DPSCs is another key step in dentin regeneration. During pulpitis, human dental pulp tissues express high levels of TNF-α and IFN-γ. TNF-α induces DPSCs odonto/osteogenic differentiation in time-dependent manner and short-term TNF-α exposure enhances odonto/osteogenic activation of DPSCs, while prolonged exposure to TNF-α diminishes the mineralization potential of DPSCs^32^,^33^,^34^. In contrast, IFN-γ induces odonto/osteogenic differentiation of DPSCs in a dose-dependent manner. Low concentrations of IFN-γ promote the proliferation and migration of DPSCs, while inhibiting odonto/osteogenic differentiation. High concentrations of IFN-γ accelerate the odonto/osteogenic differentiation of DPSCs^16^. We therefore propose that TNF-α and IFN-γ play a synergistic and interactive role in regulating dentin regeneration. At early stages of inflammation, IFN-γ at relative low concentration may promote the proliferation of DPSCs and their migration to injury sites, subsequently TNF-α induces odonto/osteogenic differentiation of DPSCs at the lesion site. With increasing severity of inflammation, the accumulation of IFN-γ further contributes to odonto/osteogenic differentiation of DPSCs. Additionally, in the period of chronic inflammation of pulp, odontoblasts and DPSCs have the ability to constantly form dentine in response to long-term irritation, such as dental caries, restorations breaking down, traumatic tooth injuries, and chemical substances^35^. This enables the healthy pulp to partially compensate for the loss of enamel or dentine. However, excessive odonto/osteogenic differentiation can lead to the pulp spaces of teeth decrease in size, as well as the loss of dental pulp cells through the deposition of secondary and tertiary dentine. As a result, the pulp may become vulnerable to injuries^35^. Therefore, the data above may provide a promising treatment by controlling the concentration of IFN-γ to inhibit the excessive mineralization of DPSCs.

We have further characterized the relevant signaling pathways controlling IFN-γ induced odonto/osteogenic differentiation of DPSCs. Nuclear factor kappa B (NF-κB) signaling is found to be active in many inflammatory diseases, such as arthritis, gastritis and pulpitis^36^. Previous studies have demonstrated that NF-κB signaling is involved in regulating odonto/osteogenic differentiation of DPSCs^37^,^38^. For example, the mineral trioxide aggregate (MTA), a biocompatible material widely applied to treat the endodontic disease (pulp and periapical infections), can promote the odonto/osteogenic differentiation of DPSCs from inflammatory sites via the activation of NF-κB signaling^39^. Additionally, TNF-α, a major inflammatory cytokines in inflammatory pulps, can trigger odonto/osteogenic differentiation of human DPSCs via the NF-κB signaling pathway^40^. In this study, we found that IFN-γ up-regulated the phosphorylation of P65, accompanied by the inhibition of odonto/osteogenic differentiation of DPSCs, whilst PDTC, a specific inhibitor of the NF-κB signaling pathway significantly suppressed the activation of p-P65 and also rescued the odonto/osteogenic differentiation capacity of DPSCs, indicating that NF-κB signaling plays an important role in the IFN-γ regulated odonto/osteogenic differentiation of DPSCs.

Our findings also demonstrate that the NF-κB signaling pathway is not the only pathway involved in the IFN-γ-induced odonto/osteogenic differentiation of DPSCs, since IFN-γ also down-regulated the phosphorylation of p38 which is associated with p38-mitogen-activated protein kinase (MAPKinase) signaling. It has been demonstrated that the p38-MAPKinase pathway is associated with odontoblast stimulation during tertiary dentinogenesis through both p38 phosphorylation and enhanced nuclear translocation^41^. Additionally, TNF-α treatment activates the p38 pathway in dental pulp cells via phosphorylation of p38 and inhibition of p38 diminishes the expression of DPP and DSP^38^. MTA can induce odontoblastic differentiation of human DPSCs via activation of MAPK signaling^42^. In a previous study, we demonstrated that LPS can promote the odontoblastic differentiation of human DPSCs via MAPK signaling pathway^19^. We herein further found that blockade of the p38-MAPKinase pathway with a specific inhibitor, SB203580 resulted in significant rescue of IFN-γ-induced odonto/osteogenic differentiation of DPSCs, indicating p38-MAPKinase signaling plays a key role in the repair responses of dentin-like complexes.

To further confirm the effects of IFN-γ on dentin regeneration *in vivo*, IFN-γ-induced DPSC/NF-gelatin scaffold constructs were subcutaneously implanted in nude mice for 4 weeks. NF-gelatin matrix which mimics the chemical composition and physical architecture of the collagen in dental matrices provides an excellent microenvironment to support DPSC adhesion, proliferation, differentiation, and new tissue formation^43^. Based on the *in vivo* results, a newly formed dentin-like matrix filling the pores of the NF-gelatin scaffolds was observed in the non-induced DPSC/NF-gelatin scaffold group. In contrast, pretreatment with IFN-γ significantly suppressed the formation of dentin-like matrix by the DPSCs and the co-treatment with PDTC or SB203580 restored the formation of dentin-like matrix, which is consistent with our *in vitro* results. Numerous studies have reported that the regenerating hard tissue by DPSCs is not always dentin-like tissue, but a bone/cementum-like structure (referred to as “osteodentin”)^44^,^45^. Although new formation of osteodentin cannot function as the real dentin, it can prevent the pulp tissues from external stimulation. The direct pulp capping is a successful clinical treatment in which a protective material (such as calcium hydroxide) is applied to exposed vital dental pulp tissues to induce the closure of the exposed site with new hard tissue^46^,^47^. We herein provide evidence that controlling signaling pathways, including p38-MAPKinase signaling and NF-κB signaling, can effectively restore the odonto/osteogenic differentiation potential of DPSCs and promote dentin-like matrix formation in an inflammatory microenvironment. This may provide a promising new therapeutic strategy for dentin-pulp tissue engineering for future endodontic treatment. Additionally, DPSCs seeded in a new developed S-scaffold which integrate the low and high-stiffness gelatin matrices into a single scaffold has been demonstrated to successfully regenerate pulp-dentin like tissue^48^. Therefore, further in-depth investigation shall be performed to evaluate the effect of IFN-γ induced DPSCs in the new developed S-scaffold.

## Conclusion

This study demonstrates that low concentrations of the inflammatory cytokine IFN-γ can stimulate the proliferation and migration of human DPSCs, but inhibits their odonto/osteogenic differentiation via NF-κB (p65) and MAPK (P38) signaling pathways. Furthermore, our *in vivo* data confirms the effects of IFN-γ on the regulation of odonto/osteogenic differentiation of DPSCs, as well as the underlying signaling pathways involved in this process. This study represents a significant advance in better understanding the regulatory effects of IFN-γ on the behavior of DPSCs, as well as providing a promising therapeutic strategy for dentin-pulp tissue engineering for future endodontic treatment.

## Material and Methods

### Cell Cultures

The clonal populations of DPSCs were isolated as previously described^19^,^49^. Briefly, pulp tissue was obtained from third molars (donors aged from 17 to 20 years) at the School of Stomatology, Fourth Military Medical University with the patient’s informed consent and Institutional Review Board (IRB) from the Stomatological Hospital of the Fourth Military Medical University approved the research project (permission number IRB-REV-2014-018). All the methods in the study were carried out in accordance with the approved guidelines. Pulp tissues were digested with a 4 mg/mL solution of collagenase/dispase for 1 h at 37 °C. Following centrifugation and resuspension in alpha modification of Eagle’s medium (α-MEM) supplemented with 100 units/mL penicillin, 100 mg/mL streptomycin, 20% foetal bovine serum (FBS) (Life Technologies, USA), and 100 μM l-ascorbic acid 2-phosphate (Sigma, USA), a single cell suspension was obtained by passing through a 70 *μ*m pore mesh cell strainer (BD Biosciences, USA). The clonal populations of DPSCs were isolated using a limiting dilution protocol, and cells at the third or fourth passages were used for this study^50^.

### Flow cytometry

The cells were washed and resuspended in PBS supplemented with 3% FBS that contained saturating concentrations (1:100 dilution) of the following reagents: FITC-conjugated anti-human monoclonal antibodies, anti-CD146-phycoerythrin (PE), anti-CD90-PE, anti-CD105-PE, anti-CD29-PE, anti-CD34-PE or anti-CD45 (PE) for 1 h at room temperature in the dark. As a negative control, PE-conjugated nonspecific mouse IgG1 were substituted for the primary antibodies. The cell suspensions were washed twice, resuspended in 3% FBS/PBS and analysed with a flow cytometry cell sorting Vantage cell sorter (Becton & Dickinson). The data were analysed with a Mod-Fit 2.0 cell cycle analysis program (Becton & Dickinson).

### Cell growth analysis using MTT

To evaluate the effects of IFN-γ on the growth rate of DPSCs, cells were treated with a range of concentrations of IFN-γ for 1, 3, 5, 7 days. DPSCs were seeded into 96-well plates at concentration of 1 × 10^3 ^cells/well. At 60% confluence, cells were serum starved for 24 h and treated with different concentrations of IFN-γ. At the indicated time points, the proliferation rates of cells were assessed with the MTT test as previously described^19^.

### 5-ethynyl-2′-deoxyuridine (EdU) incorporation assay

For proliferation studies, starved DPSCs were treated with a range of concentrations of IFN-γ for 3 days. Subsequently, proliferating cells were evaluated using the Cell-Light™ EdU Apollo^®^567 *In Vitro* Imaging Kit (RiboBio, China) according to the manufacturer’s protocol. The proliferation ratio is calculated as described previously^51^,^52^,^53^,^54^. Briefly, EdU is a nucleoside analog of thymidine that is incorporated into DNA during active DNA synthesis only by proliferating cells. After incorporation, Image-J software was used to calculate the percentage of EdU-positive cells (identified by Apollo^®^567 fluorescence) in total cells (identified by Hoechst33342 nuclei staining). The EdU positive rate was calculated in three independent assays for each sample.

### Real Time PCR Analysis

DPSCs were serum starved for 24 h and treated with a range of concentrations of IFN-γ for 3 days. Total RNA was extracted from DPSCs using TRIzol reagent. One microgram of total RNA was used as a template to synthesise first-strand complementary DNA using oligo-dT priming with the Omniscript RT kit (Qiagen, USA). QPCR were performed with SYBR Green PCR master mix reagent (Takara, Japan) in an ABI Prism 7500 qPCR system (Advanced Biosystems, USA) as previously described^3^. The human gene-specific primers for cDNA can be found in Table 1.

### Transwell Migration Assay

The chemo-migration assay was performed using transwell chambers (pore size 8 μm, Costar, USA) which were inserted into 24-well plates. 600 μL α-MEM containing a range of concentrations of IFN-γ was placed in the lower chamber and 5 × 10^4^ cells in 150 μL of α-MEM in the upper chamber. After 24 h, the medium and non-migratory cells in the upper chamber were removed gently with a cotton swab. The migratory cells on the lower side of the membranes were fixed with 4% paraformaldehyde (PFA) and stained with crystal violet. Images were captured at X10 magnification using an Olympus photomicroscope. Cells from six different fields were counted and used for analysis.

### Wound healing Assay

DPSCs were grown to 90% confluency in 100 mm culture dishes. Cells were then serum starved in 0.1% FBS for 24 h. Medium was changed for the indicated IFN-γ concentrations, and a scratch was made in the cultures using a 200 μL pipet tip. The initial scratched areas were uniform across the different samples and permanently marked; the marked areas were imaged at 12 h after scratching using an Olympus photomicroscope. The migration of the cells was determined by measuring the distance between the wounded edges using Image-J software. Three different areas were measured for each group and averages determined.

### Oil Red O Staining

For adipogenic induction, the cells were seeded in 6-well plates, grown to subconfluence, and incubated in adipogenic medium containing 1 mM dexamethasone, 0.2 mM indomethacin, 0.01 mg/mL insulin and 0.5 mM isobutyl-methylxanthine (IBMX) (all from Sigma), and 10% FBS for 5 weeks. The cells were fixed in 4% PFA in PBS and washed with 70% ethanol, and the lipid droplets were stained with 0.3% (w/v) Oil-Red O (Sigma)/60% isopropanol reagent for 60 min and then washed with water.

### Alizarin Red Staining

DPSCs were seeded into 24-well plates at a density of 1 × 10^5^ cells per well. After cells reached 80% confluence, the culture medium was changed for odonto/osteogenic differentiation induction medium with or without IFN-γ and then cultures were continued for another 2 weeks.

To investigate the related signaling pathways involved in the IFN-γ-regulated odonto/osteogenic differentiation of DPSCs, DPSCs were incubated with odonto/osteogenic differentiation induction medium containing IFN-γ 0.5 ng/mL with or without the NF-κB inhibitor (PDTC, Sigma, USA) or MAPK inhibitors (SB203580, U0126, SP600125, all from *In vivo* Gen, USA) for 2 weeks. The induction medium was changed every 3 days. Finally, cells were stained with alizarin red (pH = 4.1) and staining quantified according to previously published methods^55^.

### Western blot Analysis

DPSCs were cultured for 14 days in the odonto/osteogenic induced medium with IFN-γ supplemented with inhibitors, PDTC, SB203580, U0126 or SP600125, respectively. DPSCs cultured in the odonto/osteogenic induced medium alone for 14 days were used as the control group. As for the investigation of signaling pathways as described above, DPSCs were serum starved for 24 h and exposed to IFN-γ 0.5 ng/mL for 0, 15, 30, 60 min or co-stimulated with IFN-γ 0.5 ng/mL with one of the signaling pathway inhibitors for 60 min.

Western blot assays were performed as described previously^3^. The primary antibodies were ALP, DSPP, OCN, β-ACTIN from Santa Cruz, ERK1/2, p-ERK1/2, JNK, p-JNK, P38, p-P38, P65, p-P65, and GAPDH from Cell signaling. The relative protein expression intensities were quantified by densitometry using Quantity One analysis software.

### *In vivo* subcutaneous implantation of scaffolds

The animal experiments were performed according to the guidelines of the Animal care committee of the Fourth Military Medical University, and the IRB for Human Subjects Research of the Fourth Military Medical University approved the experimental protocols (permission number IRB-REV-2014-018). NF-gelatin scaffolds with a porosity of 96.5 ± 0.2% and macropores ranging from 250 to 420 μm were kindly provided by Dr. Tiejun Qu as described previously^48^.

DPSCs were seeded onto each scaffold. The cell-scaffold constructs were cultured in α-MEM supplemented with 10% FBS for 24 h at 37 °C on an orbital shaker (Orbi-shaker™CO_2_, USA) in an incubator with 5% CO_2_. The cell-scaffold constructs were exposed to odonto/osteogenic medium with or without IFN-γ supplemented with PDTC or SB203580 for 1 week before subcutaneous implantation. Immuno-compromised female mice (6–8 weeks old) were used for the implantation studies. One mid-sagittal incision was made on the dorsa, and two subcutaneous pockets were created using blunt dissection. Four samples were implanted for each group, where each mouse received two implants at random. After placement of the scaffolds, the incisions were closed with sutures. The animals were sacrificed and samples retrieved after 4 weeks of transplantation. The harvested specimens were immediately fixed in 4% PFA for 24 h.

All the specimens (n = 4 in each group) were analysed by micro-CT (eXplore Locus SP micro-CT; GE Healthcare, USA) to evaluate structural and mineral changes within the specimens. To determine the 3D micro architectural properties, specimens were evaluated using analysis software (MicroView; GE Healthcare). All scans and calculations were performed by the same investigator in the blind manner.

After decalcification in 17% ethylenediamine tetra-acetic acid, the samples were processed for histological observation by H&E, Masson’s trichrome and Van Gieson staining as previously described^48^.

### Statistical Analysis

Each experiment was performed at least in triplicate, unless otherwise stated. Data are reported as the mean ± SD (standard Deviation). The significance of the differences between the experimental and the control groups was determined by using one-way analysis of variance. A value of P < 0.05 was considered to be statistically significant.

## Additional Information

**How to cite this article**: He, X. *et al*. IFN-γ regulates human dental pulp stem cells behavior via NF-κB and MAPK signaling. *Sci. Rep.* **7**, 40681; doi: 10.1038/srep40681 (2017).

**Publisher's note:** Springer Nature remains neutral with regard to jurisdictional claims in published maps and institutional affiliations.

## Supplementary Material

Supplementary Figure 1

## Figures and Tables

**Table 1:** Human primer sequences used for real-time PCR analysis.

Human Genes	Primers	Sequences
GAPDH	Forward Primer	5′-CCTGCACCACCAACTGCTTA-3′
	Reverse Primer	5′-GGCCATCCACAGTCTTCTGAG-3′
Cyclin B1	Forward Primer	5′-AATGAAATTCAGGTTGTTGCAGGAG-3′
	Reverse Primer	5′-CATGGCAGTGACACCAACCAG-3′
Cyclin D1	Forward Primer	5′-ATGTTCGTGGCCTCTAAGATGA-3′
	Reverse Primer	5′-CAGGTTCCACTTGAGCTTGTTC-3′
PCNA	Forward Primer	5′-GGCCGAAGATAACGCGGATAC-3′
	Reverse Primer	5′-GGCATATACGTGCAAATTCACCA-3′
P21	Forward Primer	5′-CATGTGGACCTGTCACTGTCTTGTA-3′
	Reverse Primer	5′-GAAGATCAGCCGGCGTTTG-3′
