# Supplementary material for: IFN-γ regulates human dental pulp stem cells behavior via NF-κB and MAPK signaling
Source: Sci Rep. 2017 Jan 18;7:40681. doi: 10.1038/srep40681 (PMC5241669; doi:10.1038/srep40681)
Supplement: Supplementary Figure 1 [file srep40681-s1.pdf]

IFN-γ regulates human dental pulp stem cells behavior via NF-κB and MAPK signaling

Xinyao He DDS, MS[1,2a], Wenkai Jiang DDS, PhD[1,2a], Zhirong Luo, DDS, MS[1,3a], Tiejun Qu, DDS, PhD[1,2], Zhihua Wang, DDS, MS[1,2], Ningning Liu, DDS, MS[1,2], Yaqing Zhang, DDS, PhD[1,2], Paul R. Cooper PhD[4], Wenxi He, DDS, PhD[1,2]*,

1. State Key Laboratory of Military Stomatology, Department of Operative Dentistry & Endodontics, School of Stomatology, the Fourth Military Medical University, Xi'an 710032

2. Shaanxi Key Laboratory of Stomatology, School of Stomatology, the Fourth Military Medical University, Xi'an 710032

3. Department of Stomatology, the Affiliated Hospital of Guizhou Medical University, Guiyang 550004

4. Oral Biology, School of Dentistry, University of Birmingham, B4 6NN, UK

a These authors contributed equally to this work.

* Corresponding Author: Wenxi He, No.145 Changle West Road, Xi'an, Shaanxi, 710032, P. R. China. Tel.: +86-29-84776476 (o), Fax: +86-29-84776476 (o);

E-mail: hewenxi@fmmu.edu.cn;

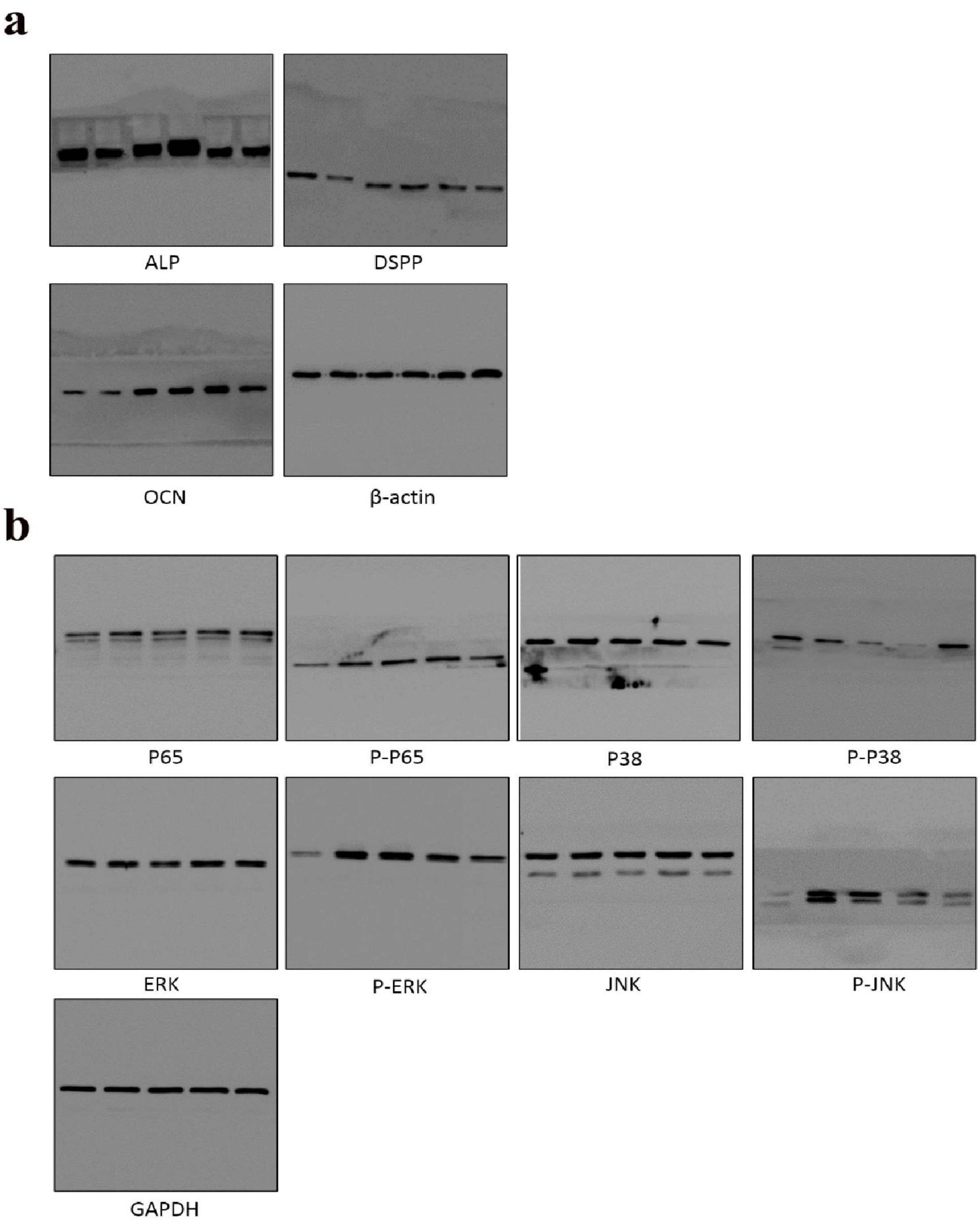

Supplementary Figure 1. Full blots and gels for Figure 4 and Figure 5. Full blots and gels of ALP, DSPP, OCN, β-actin (a), and P65, p-P65, P38, p-P38, ERK, p-ERK, JNK, p-JNK, GAPDH (b).
